# Clinical Illustrations of the More Important Diseases of Bengal, with the Result of an Inquiry into Their Pathology and Treatment

**Published:** 1835-07-01

**Authors:** 


*Clinical Illustrations of the more important Diseases of Bengal, with the Result of an Inquiry into their Pathology and Treatment.* By William Twining, m.r.c.s., first Assistant Surgeon, General Hospital, Calcutta.—Calcutta, 1832. 8vo. pp. 705.

This work will be eminently useful to those who practise in Bengal, and will be read with profit by those who have little chance of seeing any patients excepting *intra quatuor maria.* Mr. Twining is a straight-forward, sensible practitioner: he has always a distinct object in view in his treatment, has plenty of patients, keeps ample notes, and makes careful dissections. The book of such a man must be useful; but it would have been far more so, had Mr. Twining been conscious that many excellent writers had preceded him in discussing the diseases of which he treats. Instead of this, he imagines, as we find from the last sentence of his preface, that the laborious and accurate observation of facts began only of late years: "a system, of late years, happily substituted for the vague conjectures of former ages." (P. xxiv.)

Now it happens that the remedy which he recommends so strongly in the treatment of dysentery, ipecacuanha, was used with great success by a host of physicians, who lived in the ages of vague conjectures; as Mr. Twining may see by consulting Dr. Copland's Dictionary, art. *Dysentery.* We may mention Helvetius in particular, who received a large reward from Louis XIV., for introducing the use of ipecacuanha in this disease into France.

The first chapter of Mr. Twining's work is on Dysentery. After remarking that it is much more rapid and fatal in Bengal than the disease of the same name in England, he observes, that

"The most remarkable circumstances connected with the dysentery of Bengal, are the extensive local inflammation of the mucous membrane of the great intestines, coeval with the commencement of the disease; the early existence of ulceration; and, in many cases, a tendency to sloughing of that membrane; while the degree of pyrexia, and other constitutional symptoms, are for the most part incommensurate with the existing local affection, the general disorder being apparently infinitely less than is often observed attending a much slighter degree of local disease in other climates." (P. 1.)

The following are the symptoms, and a part of the post-mortem appearances:

"We generally find the ordinary dysentery of Bengal to begin as a common purging, the evacuations differing little from the character of healthy stools, except in being copious and fluid; uneasiness and griping pains in the belly soon succeed, and are followed by tenesmus; blood, with mucus, is observed in the stools, often forming the greater part of them; and pressure over the course of the colon gives pain. There is anxiety and restlessness, the pulse is little affected, the tongue often moist and white, occasionally quite clean and moist; hardly any pyrexia exists, though the thirst is commonly urgent. The severity of the symptoms increases gradually, though sometimes very rapidly, until the evacuations consist entirely of blood and slime, or of a bloody water, like the washings of raw meat. In such severe cases of only a few days' duration, masses of sloughing membrane are voided.

"Sometimes dysentery comes on suddenly, the most violent symptoms arising within thirty-six hours, and not preceded by any previous evident disorder; pure blood being poured out from the bowels in large quantities, at an early period of the disease which is attended with little distress, except the disturbance from frequent calls to rise to stool. In three or four days, the stools have a horrid odour of putrid blood, which has been compared to the smell of an anatomist's macerating tub; and there is a fœtid cadaverous exhalation from the patient's body. This odour of the stools, a rapid weak pulse, and hiccup, are almost always signs of a fatal termination. These extreme symptoms are often found to depend on numerous distinct circular ulcers in the colon, with elevated thick and abrupt edges, which are in a sloughing state; while the muscular fibres of the intestine are apparent at the bottom of the ulcer, as if dissected clean. The disease seems to commence so suddenly, that we might suppose an extensive affection of the mucous membrane of the colon to take place, much in the way that eruptive diseases of the skin arise, and ulceration follows, the sloughing edges of the ulcers pouring out copious discharges of blood: so that the patient dies from the hemorrhage and irritation, in four, five, or seven days. Notwithstanding this dreadful state of disease, there is often little or no pyrexia, the tongue does not shew much sign of disorder, the pulse is frequently soft and compressible, the skin cool and perspiring freely; and pressure on the belly gives little uneasiness until we examine with care over the cœcum, and then the patient almost always feels pain.

"In other cases, from the very commencement of the disease, the desire to go to stool is incessant, and attended with urgent straining while on the commode, the patient being obliged to rise ten times in an hour, and having very scanty evacuations from the bowels, which consist of slime and blood, without any feculent appearance; the pulse is rapid, often at the same time small and hard. An uneasy sensation above the pubis, pain in the bladder, and suppression of urine, frequently attend the worst cases of this sort; these symptoms arise from irritation extending to the lower portion of intestine, contiguous with the fundus and back of the bladder. Anxiety and restlessness increase early in the course of this form of the disease, which in its latter stages is often attended with more pyrexia than the cases already described; and the patient dies miserably emaciated between the eighth and twelfth day. In some protracted cases, which are approaching a fatal termination, the tongue becomes covered with brown mucus, or it is dry, and the teeth loaded with sordes; delirium and low fever existing at the same time.

"The above symptoms depend on the different stages of inflammation, ulceration, or sloughing of the mucous membrane of the great intestines, and the consequences thereof. Dysentery may occur during various morbid states of the constitution, such as the scorbutic, or splenic cachexia; and may be attended by various coexistent diseases.

"On dissection we find the following appearances:

"1st. Inflammation, ulceration, and at times sloughing or mortification of the inner coats of the intestines; principally affecting the cœcum, colon, and rectum.

"2d. Morbid vascularity of the mesocolon, mesentery, and omentum; adhesions of the omentum to the parts adjacent, and of contiguous portions of intestine to each other. The latter usually only happening when ulcers of the intestine have nearly perforated through the whole of its coats, and a breach that would admit of effusion of fæces into the abdominal cavity, is thus prevented.

"3d. Glands of the mesentery and mesocolon often enlarged, sometimes inflamed, and more rarely suppurating; the corresponding portion of intestine usually contains a deep and large ulcer.

"4th. The omentum is occasionally found adhering to these diseased glands, forming a band that may strangulate a portion of intestine, and cause death.

"5th. The ulcerations within the great intestine, are generally most numerous, and most extensive at the cœcum, and first portion of colon: the valvula ileo-colica, has in some cases, been found quite destroyed by ulceration, and the lower end of ilium has formed an intus-susception into the cœcum; and becoming there strangulated has caused death. In a few more fortunate instances of intus-susception, when the lower portion of ilium descends into the cœcum, and forms a circumscribed tumor in that region, attended with suppression of stools, and rapid pulse; which prove the obstruction of the canal at that part: the strangulated portion sloughs off, after adhesive union of adjacent parts has taken place, so as to maintain the continuity of the canal; and then stools are again passed, together with masses of slough, or entire portions of intestinal tube, and the patient slowly recovers. In eight years I can mention five cases of this sort, two of which have recovered.

"6th. The right portion of the omentum is frequently found adhering to the cœcum, and this morbid attachment gives rise to symptoms that are liable to be mistaken for hepatic abscess. When these adhesions exist, we find that irritation or distention of cœcum, or pressure over that part, produces pain at the transverse portion of colon, which is drawn downwards by this attachment to the part most diseased; the patient cannot stand erect, nor extend the body as he lies down, without feeling pain, which is referred to the region of the liver: the same pain is excited by raising the right arm above the head; there is occasionally cough, and sometimes a pain in the right shoulder, rendering the diagnosis very difficult."* (P. 4.)

In a few instances the size of the intestine is increased by thickening of its coats; in others, again, the whole of the great intestines are contracted in diameter. The three or four inches of ilium nearest the cæcum are generally affected with superficial ulcerations and roughness; but, with this exception, the small intestines are rarely diseased in true dysentery. In chronic cases, the cellular structure at the root of the mesentery and mesocolon is often found indurated to a certain degree, and quite void of fat. "If chronic pains in the loins and lower extremities ever depend on this morbid condition, there would be little hope of benefit to those pains, from the remedies commonly advised for rheumatism." (P. 13.)

Severe cases of acute dysentery, in plethoric patients, are to be treated by venesection, leeching, and the tepid bath.

"With this system of depletion, a dose of castor-oil is given immediately after the first bloodletting; and, when it has operated freely, the patient is made to take six grains of ipecacuanha powder, with four grains of extract of gentian, and five grains of pil. hydrarg., in three pills, which are repeated every night and morning; and twenty grains of powdered jalap, with forty grains of cream of tartar, are given daily at eleven o'clock in the forenoon. I rarely deviate from these remedies, except by occasionally using calomel in place of the blue pill; and that is not very often done. In some patients, who have recovered from the more acute symptoms for many days, and have become emaciated, with dryness of the skin, I have, after a few days, omitted the purgative of compound jalap, and ordered a drachm of sulphur, mixed with half an ounce of mucilage and one ounce of cinnamon water, to be taken in the morning early; giving the ipecacuanha, gentian, and blue pill, at four and nine P.M. In such cases the sulphur is a mild aperient: it has the property of acting on the skin, and, I believe, one of its effects in chronic dysentery is produced by its actual contact with the ulcerations of the intestines, inducing them to heal. Injections of cold water have the most certain and quickest effect in removing the painful affection of the bladder, with suppression of urine, that attends bad cases. It has been many times

* "Staff Surgeon Marshall has stated, that the most frequent adhesions of the omentum are to the cœcum; and Mr. Annesley says, that the omentum is often adherent to the brim of the pelvis: but neither of these authors has adverted to the direct influence of those adhesions, in causing a pain at the epigastre, or at right portion of the colon and edge of right false ribs, which is liable to be mistaken for liver disease."

found an excellent remedy in cases where copious discharges of pure blood take place. A solution of ten grains of sugar of lead, in ten ounces of cold water, is often of great effect in the like circumstances; and, where tenesmus is severe at night, sixty drops of laudanum, with two ounces of cold water, given as an injection, has often remained in the rectum all night, and procured excellent rest." (P. 14.)

The use of mercurials is not to be pushed to salivation.

Tenesmus often depends on ulceration low down in the rectum, and may be relieved by acetate of lead, in the form of ointment. Blisters are rarely advisable in the commencement; and opium is very seldom useful. The diet should, of course, be extremely low. Slight cases may be cured by low diet, a daily dose of castor oil, and two or three nauseating doses of ipecacuanha, or antimonial wine, daily. An infusion of ipecacuanha and ginger, and a diet consisting solely of a teacupful of barley-water, three times a day, have often cured slight cases.

The following are our author's first two cases, or, as he calls them, by an uncommendable Gallicism, *observations*.

"*Observation* I. Thomas Bullery, æt. thirty-eight, a stout man, of light complexion, arrived from England six weeks ago: he had been in India before, and has been now living on board ship. Was attacked with dysentery on the 18th October, 1829, and his complaints increased daily until the 25th, when he was landed in the evening, and sent to the General Hospital. The belly was then rather full and inelastic; pressure over the course of the colon caused pain, but there was very little pyrexia: he stated that his evacuations were very frequent and scanty, consisting mostly of blood, and that he was up to stool twenty-four times last night.—V.S. ad lb. jss. ℞. Calomel, gr. xii.; Extract. Hyoscyami, gr. iv. in two pills at bedtime.*

"October 26th. Blood not buffy. He has been up to stool very often, but voided nothing. He had a slight rigor in the night; there is no pyrexia now; pulse eighty; tongue clean at edges, with white mucus on its centre. The belly is rather full, doughy, and inelastic; pressure across the navel causes pain.—Apply twenty leeches to the belly immediately. Let him take

* Mr. Twining says, with great *naïveté*, "In the course of this work, the directions appear in English, under the Latin prescriptions; which is the custom followed here, in the hospital diaries, for the purpose of preventing mistakes in the administration of the medicines by the young apprentices; and I have not thought it important to make any alteration." (*Pref.* p. x.) We confess that we are among those who, in a case of this kind, would rather (to use parliamentary language,) that the qualification was elevated to the level of the trust, than the trust lowered to the level of the qualification.

Pulv. Jalap. comp. ℈j. at seven in the morning. Tepid bath at noon, and give Olei Ricini ℥j. after the bath.

"Vespere. Had twelve free stools, with very little blood, and he is better.—℞. Pil. Hydrarg., Extract. Colocynth. comp. aa gr. iv. at bedtime.

"Oct. 27th. He had only three stools in the night, and is better in every respect.—Pulv. Jalap. comp. ʒj. immediately.

"Oct. 28th. Had five stools in the day: none at night.—Medicine repeated.

"Oct. 29th. Convalescent. No more medicine used. Discharged well on the 2d November, 1829.

"*Obs.* II. Henry Pritchard, æt. twenty-one, a middle-sized man, of dark complexion, recently arrived from England, was taken ill with dysentery on the 20th November, 1830, and became gradually worse every day, till he was sent to hospital on the evening of the 25th. He stated that he was passing much blood with his stools, which were attended with very distressing tenesmus. The belly was full and hard, face flushed, pulse ninety-two and full.—V.S. ad lb. jss. ℞. Calomel, Extr. Colocynth. comp. aa. ℈ss., in pills at bedtime.

"November 26th. The blood drawn last night is buffy and cupped. He had eight stools in the night, consisting of blood and mucus, attended with dreadful tenesmus. The belly is hard and hot; the tongue moist, and loaded with brownish mucus. The fever and flushed face continue. Pulse ninety-six, and full.—V.S. ad lb. jss. immediately. ℞. Pulv. Jalap. comp. ʒj. at seven A.M. Apply twelve leeches to the belly at noon. ℞. Pulv. Ipec. gr. xii.; Extract. Gentian. gr. viij.; Pil. Hydrarg. gr. x. Misce et divide in pil. No. vj. Three pills to be taken at noon, and three more at bedtime.

"Nov. 27th. Had twenty-four stools in the night, consisting of fæces mixed with slime and blood: he has suffered much from tenesmus and straining. His belly is hard and hot, and some pyrexia remains; but the pulse is eighty, and soft.—V.S. ad lb. j. immediately. Sixteen leeches to the belly at noon. Medicine repeated as yesterday. Laudanum, ʒj. to be given in two ounces of cold water, as an enema, at bedtime.

"Nov. 28th. Blood florid, and not buffy: he had five stools in the day, consisting of blood and mucus; fourteen at night; and he is feverish.—Apply eight leeches to the belly. Medicine repeated as yesterday.

"Nov. 29th. Eight stools, (yellow fæces, and mucus without blood.) He feels easier; but the cheeks are flushed, the pulse seventy-six, weak, and soft. Some enlargement, with induration of the liver, is now perceptible.—Apply twelve leeches to the liver. Tepid bath two hours after the leeches. To take Pulv. Jalap. c. ℈j. at ten o'clock. Three of such pills as were ordered on the 26th to be given at seven A.M., and repeated at noon and at eight in the evening. Anodyne enema at bedtime, as on the 27th.

"Nov. 30th. Was frequently purged yesterday, but has had only five stools since the enema last night: the evacuations contain much mucus. He has still some pyrexia, and elastic tension of the belly.—Apply sixteen leeches to the epigastre immediately. Three pills, as on the 26th, to be taken at seven A.M. Pulv. Jalap. comp. ʒj. at noon. ℞. Extract. Colocynth. comp., Pulv. Ipecac., Pulv. Hyoscyami, Pil. Hydrarg. aa gr. iij., in pills at bedtime.—Enema repeated at night.

"December 1st. Was freely purged by the jalap; had onlv two stools in the night. The liver is still hard.—Apply eight leeches to the region of the liver. ℞. Extract. Colocynth. comp. ℈ss.; Pil. Hydrarg. gr. v., in pills at seven A.M. The anodyne enema and night-pills repeated at bedtime, as yesterday.

"Dec. 2d. Has only had five stools, which are scanty and slimy. There is no pyrexia at present. The right rectus abdominis muscle is more tense than the left.—Apply six leeches over the liver. ℞. Extract. Colocynth. comp., Pil. Hydrarg., aa gr. v., to be taken in pills at seven o'clock. Tepid bath at noon. Pulv. Jalap. comp. ʒj. after the bath. Two of the pills at bedtime, as prescribed on the 26th November.

"Dec. 3d.—Had four copious, dark, feculent stools.—Ordered to take two of the pills prescribed on the 26th November, morning and night; and compound powder of jalap ʒj. at noon.

"Dec. 4th. He had three natural loose stools last night, and his gums are sore. Belly still hard and tense, which I find is caused by his having clandestinely obtained an improper quantity of food; therefore an emetic is now ordered, and its repetition promised daily, if the belly should be tense.

"From this date he took one drachm of compound powder of jalap daily at noon, and the pills (such as prescribed on the 26th November,) night and morning, till the 10th December. After that, his bowels were kept free by Pil. Rhei c., which was given daily, till he was discharged on the 12th January, 1831." (P. 21.)

We must pass over the remainder of the chapter on Dysentery, though not without regret, and not without mentioning especially the observations on scorbutic dysentery, (in which calomel would be destructive,) and the section on "particular affections of the cæcum during dysentery." In a case belonging to this subdivision, a round earthy mass, larger than an olive-stone, was found in the centre of an abscess, which was outside and behind the cæcum, but not communicating with its cavity.

The second chapter (p. 135 to 269,) treats of Diseases of the Liver, and commences with an account of the post-mortem appearances, which is of the highest interest; but, lest this review should swell out into a volume, we must content ourselves with extracting a single passage, relating to abscess.

"Abscesses of the liver. These vary much in appearance; and their peculiarities seem in some instances, influenced by the existing diathesis of the constitution. At present, I will hardly venture to do more than enumerate the morbid conditions that have been observed."

"*a.* A large quantity of puriform matter in a cavity, the contiguous parts of the liver exhibiting not much appearance of disease. The contents of the abscess, are frequently a dark-brown, or reddish serum, and then the adjacent parts of the liver are softened and gorged with blood. The acute abscesses which form in the course of fevers, acute dysentery, and in drunkards.

"*b.* In other cases, there is only a small quantity of matter, compared with the extent of the disorganization; a considerable portion of the disease consisting in a quantity of large, tough, white or grey sloughs, hanging from the sides of the cavity, and nearly filling it: the parts on dissection much resembling the advanced stage of a large carbuncle. This disease more frequently happens in scorbutic subjects than others.

"*c.* In a few cases, we find a circumscribed abscess, the size of an orange: the matter deeply seated, and contained in a cavity, which is bounded by a thick coat of coagulable lymph.

"*d.* Numerous small abscesses, the size of a filbert, dispersed through the substance of the liver; the cavities of some, lined with a thin coat of coagulable lymph; others without any lining, appearing as if scooped out with a sharp instrument: the intervening parts of the liver soft, but not otherwise diseased. This morbid appearance is rare: it sometimes exists without much evident tumefaction of the liver. The subjects have been mostly delicate, and of scrophulous constitution.

"On inspecting the bodies of persons who have died suddenly from accidents or otherwise, I have several times met with appearances in the liver, which were considered to be the incipient or preliminary stage of abscess. These were, distinct, circumscribed, ecchymosed spots, at the concave surface of the liver; and serous interstitial effusions into the structure of that organ, near its convex surface: the latter spots, if diffused and extensive, rendering the part very soft for a considerable space. These may be considered the early changes induced by acute disease, not very rapid in its progress; and had the patients not died suddenly from other causes, these effusions would have probably terminated in abscesses, if not treated in the most judicious manner, for a long time.

"In dissection of subjects, who died of Abscess of the Liver, I have twice found a small quantity of puriform matter in the right ventricle of the heart; and in both instances, was able to trace the same appearance with small filamentous coagula, quite into the veins of the liver. The internal membrane of the hepatic veins was inflamed, but I could not discover any communication between the abscess of the liver, and these vessels; and am therefore disposed to ascribe the formation of pus, to inflammation of the hepatic veins. Both these patients were recent arrivals in India, not of very temperate habits, and their complaints began as common diarrhœa of severe description. On referring to their cases, the only difference observed between these and the usual course of Liver Abscess, was the more active pyrexia in the early part of the disease; and towards the conclusion unquenchable thirst, extreme anxiety, and frequent disposition to faint: but the patients never complained of palpitation, or any other symptom directly referred to the heart. In these cases, some degree of morbid heat of skin, continued to the last." (P. 138.)

A remarkably instructive case of acute hepatitis is narrated at p. 177 et seq. The patient was cured by leeching, purging, and starving; the last remedy being carried to an extent which does great credit to the resolution of the patient, as well as the clear-sightedness of his doctor. For a whole week "he was allowed a cup of tea night and morning, but no food, and no drink in large quantity: only a table-spoonful of water every half-hour, if thirsty." (P. 180.)

Mr. Twining informs us, that rigor has not occurred in the majority of cases of abscess of the liver which he has seen.

In one instance the abscess was successfully opened:

"The proportion of patients who recover, after the formation of an extensive Abscess of the Liver, in Bengal, is lamentably small. I have as yet seen only one case in which abscess of the liver was opened by an incision. In that case the opening was made near the epigastre; the patient recovered, and lived by no means a temperate life afterwards. On his death, which occurred many years after, from causes unconnected with liver affection: I opened the body, and found adhesions of the convex surface of the liver anteriorly, and an extensive thick fibrous structure, occupying a space at that part, of about three inches in extent, and nearly half an inch thick. The liver rather small, of lurid brownish red colour, slightly mottled internally; the gall-bladder small, and covered with a dense false membrane." (P. 200.)

The third chapter (p. 271 to 360,) treats of Diseases of the Spleen. The following are the post-mortem appearances, placed in the order of their frequency:

"A soft rounded enlargement of the spleen, the texture less firm than in the healthy state; and easily broken if the finger be pushed abruptly against it. In some cases the part is so much softened, that it resembles a great clot of blood, wrapped in a thin membrane: this varies in colour, from black, to brown or blue; and in the extreme degree of softening, when we attempt to lift the tumid spleen, the fingers are thrust through the membrane, and the organ breaks down in the hands, becoming a putrid gore. This soft globular enlargement, from vascular engorgement of the spleen; most commonly attends, or follows, the severe remittent fever of the rains and cold season: when that disease attacks weak and unhealthy young persons.

"2. Oblong enlargement of the spleen; the organ being more firm in texture than in its natural state, its edge thin and notched: the colour being sometimes a pale brown, though more generally a dusky red. This morbid change of structure, would appear to be the result of more slow and gradual degeneration, which in its earlier stages has probably been attended with some inflammatory condition of the internal structure of the spleen: in such cases we also find evidence of superficial inflammation, attended with adhesions to adjacent parts, more frequently than in the rounded enlargement from simple vascular engorgement.

"3. Opaque patches of various sizes, some of these extend over half the convex surface of the spleen, and are nearly one eighth of an inch thick; they may be deemed the result of albuminous depositions during superficial inflammation.

"4. Adhesions of the peritoneal coat of the spleen to contiguous viscera; which adhesions are by no means a general result of tumid spleen in Bengal.

"5. In a few old cases, we find a more indurated friable spleen; that breaks when handled without much force, like a piece of old moist cheese.

"6. Still more rare, is the firmer induration intersected with septa of condensed fibrous structure; to which we give the name of scirrhus.

"7. Tubercles of various sizes, generally small, and of grey, or brown colour.

"8. An organized coagulum in the splenic vein.

"9. Encysted tumours.

"10. Abscess of the spleen.

"The four last-mentioned morbid appearances, are exceedingly rare in Bengal." (P. 280.)

The two great points in the treatment are to avoid the use of mercury, and to give iron, with bitters and purgatives.

"My usual formula for cases where there is not much pyrexia is

℞. Pulv. Jalap., Pulv. Rhei, Pulv. Calumbæ, Pulv. Zingiberis,

Potassæ Supertartratis, aa ʒj.

Ferri Sulphatis, ℈ss.

Tinct. Sennæ, ʒiv.

Aq. Menthæ Sativæ, ℥x. Misce.

This prescription is called the *Spleen mixture*. The dose is one ounce and a half for an adult, at six A.M., and repeated at eleven A.M. daily. For children, the doses are regulated so as to produce not less than three, and not more than four stools daily." (P. 283.)

Mr. Twining observes, that the disorders most closely allied to the splenic cachexiæ are chlorosis, scorbutus, and some species of anæmia. If this be so, and we see no reason to dissent from his opinion, it will give a good theoretical foundation for the practical fact, that mercurial remedies are inexpedient, and even dangerous, in these splenic maladies.

This chapter on the Spleen is by no means obnoxious to the fault with which some parts of this work are chargeable,—of omitting the opinions of other writers: on the contrary, they are often referred to, and impartially balanced.

The fourth chapter is on Cholera, (page 361 to 556.)

The following extract from the account of the varieties of cholera, with the author's method of treating them, will gratify our readers.

"If the diseases which are acknowledged to be Cholera, were so ranged on a scale as to place those cases which have most affinity, together; and those most dissimilar, at a distance: we should find one end of this scale occupied by diseases in which the actions of the constitution are distinctly febrile, and in many of them the evidence of local inflammation is strong and unequivocal, as in the most intense examples of gastro-enteritis. At the other extreme of this scale, we should find the prevailing characters of the disease, as already stated, consisting in coldness, depression of vital actions, and extreme venous congestion; with tendency to sudden death, not preceded by much active disease. Between these two extremes, every possible variety exists: the disease with early collapse and coldness, generally combines an intense degree of congestion of blood in internal organs, with some remote tendency to inflammation of the intestines, and sometimes (though rarely in Bengal,) of the brain: while the febrile cases, and those which are marked by distinct evidence of local inflammation; are by no means void of congestion, and they frequently pass suddenly into the low state, with coldness, and the most awful prostration of vital power. Our watchful attention to the course of the disease is urgently demanded, on account of this occasional tendency to sudden change; lest we be misled, and induced to use depletion, by V. S. or other means, at a time when such treatment may be injurious. It will be evident that the treatment of cholera must be varied according to the nature of the disease.

"In the febrile and inflammatory stages of the disease, attended with violent and painful spasms, warmth of surface, and free circulation, our chief dependence must be on V.S., leeches, and purgatives of calomel or blue pill, with cathartic extract, alternated with castor-oil. In a few of these febrile cases, we may venture on jalap and scammony, at more remote periods of the disease. The earlier a case of cholera of this description is bled, the more certain and effectual is the relief which is obtained. While those patients who come under treatment at a late period of the disease, even though distinctly marked inflammatory symptoms be present, require great caution in the employment of depletion; and still they are almost certain to die without antiphlogistic treatment. Opium is admissible for one or two doses, in small quantity, at the onset of these febrile cases, when watery evacuations prevail; but, except for the purpose of allaying the dreadful commotion of the system, and to arrest profuse purging, we derive little benefit from this remedy. Nothing relieves the spasms of the early stage of febrile cholera so effectually as the lancet.

"There is a more remote stage of the disease, in which local inflammations take place, appearing sometimes to be excited by premature return to a diet of animal food, and in other cases to arise without any evident cause: we are obliged then to use the lancet, and to purge the patient freely, as in an ordinary inflammatory fever. At the same time, a word of caution is requisite, lest the inexperienced practitioner should mistake for fever or inflammation the transient and ineffectual reaction which often occurs just before death, attended with morbid heat of forehead and chest, while the patient is torpid, blue, and restless; as vain attempts have been made to cure these cases by bleeding. The least that can be said of such treatment is to acknowledge its total inutility.

"Where the evacuations have been profuse, it is always advisable to give a small quantity of thin sago, or arrow-root, as soon as the stomach will retain it; and the employment of a small quantity of food of this sort need not interfere with the general antiphlogistic plan above stated.

"The majority of these febrile cases can generally be saved, if seen early, and treated with careful discrimination and perseverance." (P. 402.)

Chapter fifth treats of Fevers, (from page 557 to the end of the volume.) Like other spendthrifts, reduced to the most pinching economy, we cannot afford even the smallest extract from this division of Mr. Twining's work, and must be satisfied with giving a slight summary of some few of his opinions.

*Intermittent Fever.* Our author treats this disease by purging, bleeding in the cold stage, and quinine. Bleeding has always proved safe, and generally more successful than any other remedy: it must be performed at the commencement of the rigor, and the quantity of blood taken should not be more than from twelve to twenty ounces in a European, and from four to ten in a Bengalee.

*Continued Fever.* Bleeding, purging, and total abstinence from food, are our author's remedies in this disease. A European patient, of unbroken constitution, may be bled to lb. jss. or lb. ij.; after which, twelve grains of calomel, and twelve of cathartic extract, are to be administered; and this, again, is to be followed by a black draught six hours afterwards. The superlative frugality of the diet rises many degrees above the diæta parcissima of this part of the world. It consists of a cup of tea night and morning, and a wine-glassful of toast-water every hour. We observe that, in two cases which terminated in death, (the 135th and 136th,) hemorrhage had taken place in the brain, in spite of the most enormous bleedings. In the 135th case, 104 ounces of blood were taken by venesection in four days, besides the repeated application of leeches: in the 136th case, the amount taken by venesection was sixty-four ounces, and sixty-eight leeches were applied. Ought the bleedings to have been still more copious; or do these cases support the theory of those who maintain that bleeding does not lessen the quantity of blood in the brain?

*Remittent Fevers.* The remittent fever of the Bengal rainy season is among the most formidable diseases of India. It sometimes happens that, after two or three slight paroxysms, a change for the worse suddenly takes place, without any evident cause, and death follows within an hour. The treatment consists chiefly of two copious bleedings, a couple of scruple-doses of calomel, and leeching; and the use of quinine at a later stage. The detraction of blood, by the lancet or by leeches, must take place during the paroxysm: when this is over, the loss of blood is highly dangerous.

*The insidious Congestive Fever of the Cold Season* seems to differ from ordinary fever chiefly, if not solely, in the slowness with which the symptoms come on: a deceptive slowness, which induces the patient to trust to inefficient or pernicious domestic remedies. The treatment is the same in kind as in common continued fever, but less vigorous in degree, bearing a due proportion to the comparative mildness of the symptoms.

*Nakra.* This is a singular febrile affection, attended with swelling and inflammation of the Schneiderian membrane. It is called by the Bengalese *Nakra*, or *Nasa*, which literally means the nose disease. It never terminates in suppuration or ulceration, or in any chronic disease resembling ozæna: it is seldom, if ever, fatal, and never attacks Europeans.

We again recommend this work to the notice even of those who are not destined to practise in India. Diseases are most easily studied when of intense severity, and the treatment which they then require may be adapted to the milder forms. The entire abstinence enjoined and observed by the Bengalese in diseases attended with fever may afford a valuable hint for the management of British sick rooms.

